# Generative AI‐Enabled Discovery and Experimental Demonstration of Ultra‐Broadband Epsilon‐Near‐Zero Perfect Absorbers

**DOI:** 10.1002/advs.77966

**Published:** 2026-09-27

**Authors:** David Dang, Meena Salib, Quynh Dang, Sudip Gurung, Juan Calixto, Xuguo Zhou, Stuart Love, Massee Akbar, Aleksei Anopchenko, Wilton J.M. Kort‐Kamp, Ho Wai Howard Lee

**Affiliations:** ^1^ Department of Physics and Astronomy University of California Irvine California USA; ^2^ Center for Integrated Nanotechnologies Los Alamos National Laboratory Los Alamos New Mexico USA; ^3^ Beckman Laser Institute and Medical Clinic University of California Irvine California USA; ^4^ Eddleman Quantum Institute University of California Irvine California USA; ^5^ Theoretical Division Los Alamos National Laboratory Los Alamos New Mexico USA

**Keywords:** epsilon‐near‐zero, generative AI, inverse design, nanophotonics, physics‐informed machine learning

## Abstract

Artificial intelligence is reshaping the discovery and engineering of photonic materials. Here, we introduce a data‐driven framework that employs a generative deep neural network to create ultrathin, multilayer epsilon‐near‐zero absorbers with record‐breaking broadband performance. Within this framework, the neural network learns a generative distribution over high‐performing multilayer designs, optimizing the refractive index, layer arrangement, and thickness for maximal broadband absorption. Guided by AI, we experimentally demonstrate an optimized photonic structure that achieves near‐perfect light absorption (exceeding 98%) across more than 1000 nm bandwidth in the near‐infrared range, while remaining under 160 nm thick. Our approach demonstrates the power of integrating advanced computational algorithms with state‐of‐the‐art fabrication technology, offering a scalable route to high‐efficiency nanophotonic devices for target applications. Altogether, this work establishes a generalizable strategy for algorithm‐driven photonic design with implications across energy, sensing, and integrated optics.

## Graphical abstract

Researchers developed and applied a generative artificial intelligence (AI) model to optimize epsilon‐near‐zero thin film stacks for ultra‐broadband perfect absorption of light. The fabricated AI‐designed absorber, with a total thickness of only 160 nm, experimentally demonstrated an absorption bandwidth exceeding 1000 nm while maintaining greater than 98% absorption across the near‐infrared region.

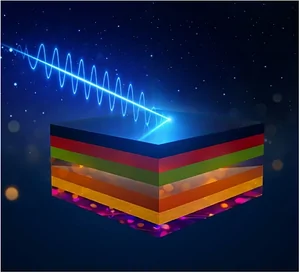

## 1 Introduction

Broadband light absorbers have a wide range of optical applications. They can be used as light filters in thermal imaging cameras, infrared sensors, and gas detectors for security and environmental monitoring. In defense applications, they reduce radar and thermal signatures, enhancing stealth technologies [1]. Additionally, they also find applications in medicine for photothermal therapy and in more efficient solar cell technologies [2]. Commercially available light absorbers use thick, alternating layers of dielectric materials or heavy ferrite‐based paints [3] to achieve their spectral properties, which often have bandwidth limitations. State‐of‐the‐art broadband absorbers with nanoscale metasurfaces can achieve broadband absorption but have limited absorption thresholds or require extensive lithography processes, which are impractical for large area manufacturing or complex geometries [4, 5, 6, 7, 8]. An emerging alternative that addresses these challenges involves multilayer thin films composed of epsilon‐near‐zero (ENZ) materials.

ENZ materials are characterized by a near‐zero real part of their electric permittivity at specific wavelengths, enabling extreme light confinement [9] and large field intensity enhancements [10] that offer new avenues to manipulate light in nanophotonic devices. For instance, ENZ materials have been proposed and employed to realize large optical nonlinearities [9, 11], high harmonic generation [8], non‐reciprocal magneto optical effects [12], and perfect absorption of light [13, 14, 15, 16, 17, 18, 19, 20]. Transparent conducting oxides (TCOs), such as indium tin oxide (ITO) and aluminum‐doped zinc oxide (AZO), are particularly attractive to the photonics community because they combine high optical transparency with electrical conductivity, resulting in their widespread use in handheld electronics and touchscreens. Beyond these conventional applications, TCOs also provide a versatile platform for ENZ photonics because their free‐carrier concentrations and mobility can be tuned through deposition parameters, including dopant ratios and temperature. This tunability allows their ENZ wavelengths to be carefully engineered across a wide spectrum [21, 22].

Using the Kretschmann‐Raether configuration, a long‐range surface plasmon ENZ mode can be excited in thin ENZ films [9, 23, 24]. This excitation enables near‐perfect absorption of light close to the ENZ wavelength, depending on light's incidence angle inside the prism. However, while the absorption is strong (≥98%), the bandwidth is extremely narrow (a few nanometers). Broadband absorption, spanning hundreds of nanometers, can be achieved by stacking multiple layers of ENZ materials [13, 17]. Yet, previous multilayer ENZ absorbers exhibited limited bandwidth and did not leverage advanced machine learning with their design. Unlike single‐layer TCO absorbers, which can be optimized to operate at specific wavelengths through simple parameter sweeps, multilayer structures introduce significant complexity due to interlayer couplings and a combinatorially large parameter space. Although planar broadband absorbers can be created using homojunctions of a single TCO material with a tunable ENZ wavelength [19], achieving a truly broadband absorber requires the heterogeneous integration of different materials with ENZ wavelengths covering a broad range, from visible to infrared. A solution to exploring this vast design space is the usage of deep generative AI algorithms.

Over the past decade, deep learning has transformed a variety of scientific disciplines and industries, from image and video recognition [25] and natural language processing [26] to autonomous vehicles [27] and healthcare [28]. More recently, machine learning, particular generative artificial intelligence (AI), has been applied in nanophotonics to aid the design of metasurfaces, either by generating optimized nanostructures [29, 30, 31, 32] or serving as surrogate physics solvers for Maxwell's equations [33, 34].

In this work, we develop and validate a deep generative neural network that designs broadband perfect ENZ absorbers using material parameters constrained to the ranges achievable through well‐established thin film deposition methods, including atomic layer deposition and sputtering. In contrast to our prior design‐only study of homogeneous ITO stacks [19], the present framework optimizes a heterogeneous ITO/AZO heterostructure over an expanded set of per‐layer Drude parameters (carrier density, mobility, and effective mass), and we experimentally fabricate, characterize, and measure the resulting device (see Section S1 for a table comparison). Our experimentally fabricated multilayer broadband absorber shows over 98% absorption across a ∼1075 nm bandwidth in the near‐infrared, with a total thickness less than 160 nm. To our knowledge, this is the broadest bandwidth at this high absorption threshold for a thin‐film stack under 200 nm thick. This performance is enabled by the integration of precise fabrication control of layer thickness and material properties with machine learning‐driven optimization of the ENZ optical response. This work opens new directions for intelligent materials discovery, rapid prototyping, and functional devices that meet the demands of next‐generation energy, sensing, and optical technologies.

### 1.1 Deep Neural Network Design Approach

To design ENZ broadband perfect absorbers with high efficiency and manufacturability, we developed a generative deep neural network that decodes random input vectors to optimized physical parameters for multilayer thin‐film stacks [35]. The network generates material‐specific properties for each layer of either aluminum doped zinc oxide [36] or indium tin oxide (ITO), including carrier density, carrier mobility, and thickness. The carrier density primarily determines the plasma frequency and therefore tunes the ENZ wavelength of the material, while the effective electron mass also contributes to the plasma‐frequency response. The carrier mobility determines the damping term in the Drude model through the scattering rate and therefore controls the optical loss of the TCO film. In addition to these material parameters, the layer thickness plays an important role in determining optical interference, field confinement, and modal coupling between adjacent ENZ layers.

For fabrication considerations, the material sequence was fixed to an ITO‐first stack followed by AZO layers to satisfy fabrication constraints associated with sputtering and to prevent thermal annealing of AZO layers during high‐temperature ITO deposition. However, the model itself is capable of assigning independent material choices for each layer, and additional simulations with unrestricted layer‐wise material assignment are provided in the Section S2 (Figure S2).

**FIGURE 1:**
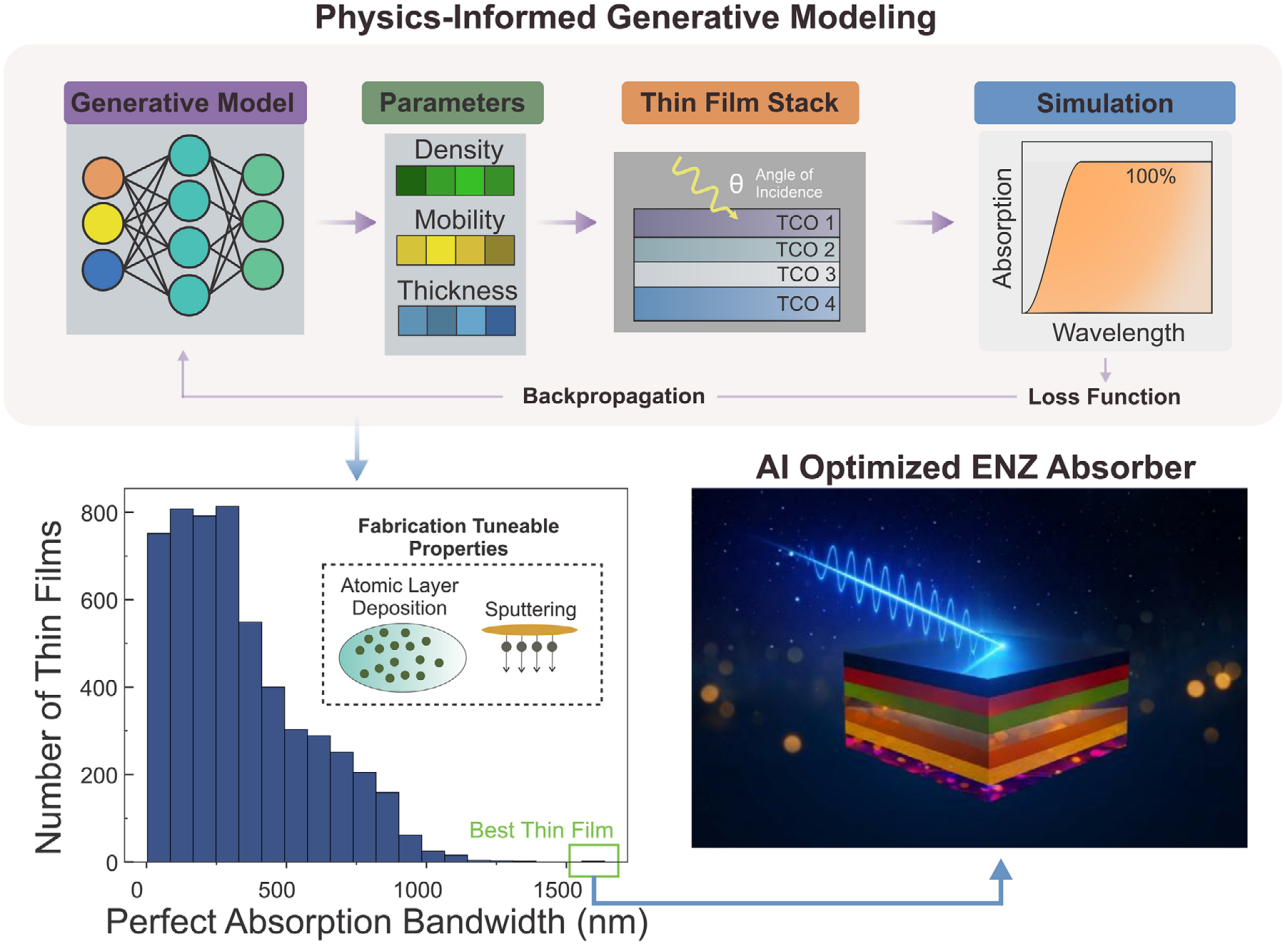
Neural network design of ENZ broadband absorber. The generative neural network chooses four parameters‐thickness, carrier density, carrier mobility, and effective mass‐each within ranges reported for ITO or AZO. The latter three parameters are used in the Drude model to compute the material's complex refractive index. The refractive index and thickness are then employed in the transfer matrix method to simulate the absorption spectrum of the generated thin film stack of TCO materials at a specified angle of incidence. Thousands of devices are generated and the best performing one is chosen for fabrication and measurement. The histogram displays the bandwidth for devices generated by the neural network achieving ≥ 98% absorption. Out of 10 000 thin film stacks generated by the neural network, 6840 met the 98% absorption criterion.

These parameters are then used to compute the corresponding absorption spectrum via the transfer matrix method (Figure 1). The target absorption spectrum was selected based on the experimentally accessible optical properties of the ITO and AZO thin films, as well as the spectral range of our measurement setup. Therefore, the optimization target was chosen to emphasize broadband absorption over the near‐infrared spectral region (800 to 3600 nm) where ENZ modes can be experimentally realized and measured. A loss function quantifies how close the simulated absorption is to the ideal absorber, and gradients are computed to iteratively improve the model's weights [37, 38], allowing the network to “Learn”.

The core architecture of our model consists of a 512‐dimensional noise vector sampled from a uniform distribution, passed through a residual neural network (ResNet) that outputs a complete thin‐film design. Residual connections help preserve gradients during training and enable deeper, more stable learning [39, 40, 41]. The model was implemented in PyTorch [42] and in order to integrate physical modeling directly into the training loop, we employed a differentiable PyTorch‐based implementation of the transfer matrix method, known as TMM‐Fast [43]. This physics‐guided approach enables the network to learn relationships between the design parameters and optical response within the sampled design space, without requiring a precomputed training dataset. (see Methods and Figure S1 for further information about model parameters).

**FIGURE 2:**
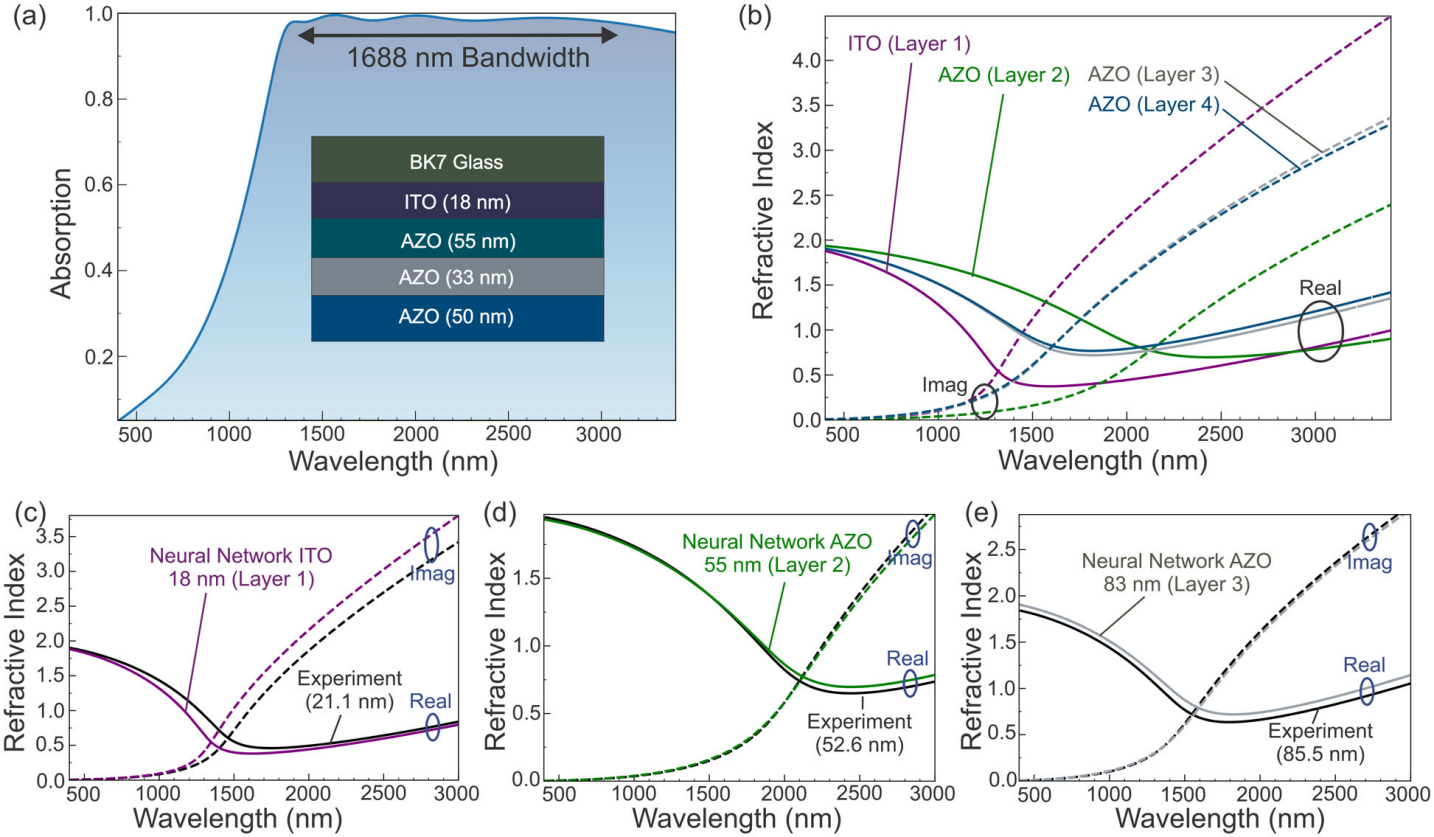
Schematic of the most broadband thin film stack designed by our generative model. (a) The total thickness is 156 nm and achieves a bandwidth of 1688 nm with simulated absorption above 98%. (b) The complex refractive index of the different layers of the absorber (solid lines represent the real part, while dashed lines represent the imaginary part). (c–e) Comparison of the refractive index from experiment vs. neural network design.

After training, the generative model was used to produce 10 000 candidate devices. Figure 1 illustrates the general architecture of the thin‐film stack and presents a histogram of the bandwidths of the generated devices. The top‐performing design achieved ultra‐broadband absorption exceeding 98% in the near‐infrared range (1500–3000 nm) with a total thickness of just 156 nm (Figure 2a). Figure 2b shows the real and imaginary parts of the refractive indices of the neural network designed individual layers. As the real part of the permittivity approaches zero, the normal component of the electric field is strongly enhanced inside the ENZ material due to Maxwell's boundary condition for the normal electric displacement field. This enhanced field strongly couples to the intrinsic losses of the ENZ materials, resulting in the perfect or near‐perfect absorption of light.

For experimental fabrication, the third and fourth layers‐having similar refractive indices‐were combined into a single layer of 88 nm thickness, effectively reducing the four‐layer design to a three‐layer configuration. We note that this simplification introduces a manual post‐processing step, rather than representing a strictly end‐to‐end AI‐designed methodology. However, TMM simulations between the original four‐layer absorber and the adjusted three‐layer design exhibit very similar optical responses, with only a 3.2% relative difference in absorption bandwidth and a 0.14% change in peak absorption (see Figure S4). These results indicate that the layer‐merging step preserves the performance of the neural‐network‐generated absorber while enabling a more practical fabrication route. To fabricate our generative AI‐designed broadband absorber, we employed atomic layer deposition (ALD) and radio‐frequency (RF) sputtering that enables tunable doping via the deposition parameters [22] (see Methods and Section S3 for all fabrication details).

## 2 Results

To measure absorption, we used a Cary‐7000 universal spectrophotometer for the reflection and transmission spectroscopy. The system contains a lamp that generates polychromatic light, which is filtered by a grating monochromator and polarizer, to select specific wavelengths and polarizations (Figure S8). Our modeled spectral range spans wavelengths from 400 to 3000 nm; however, the upper limit of the indium gallium arsenide photodetector is near 2600 nm, which restricted the experimental upper bound. The sample mount inside the spectrophotometer as well as the detector can rotate allowing us to measure reflection (R) and transmission (T) at various angles. Here, absorption is defined as *A*  =  1 − *T* − *R*.

**FIGURE 3:**
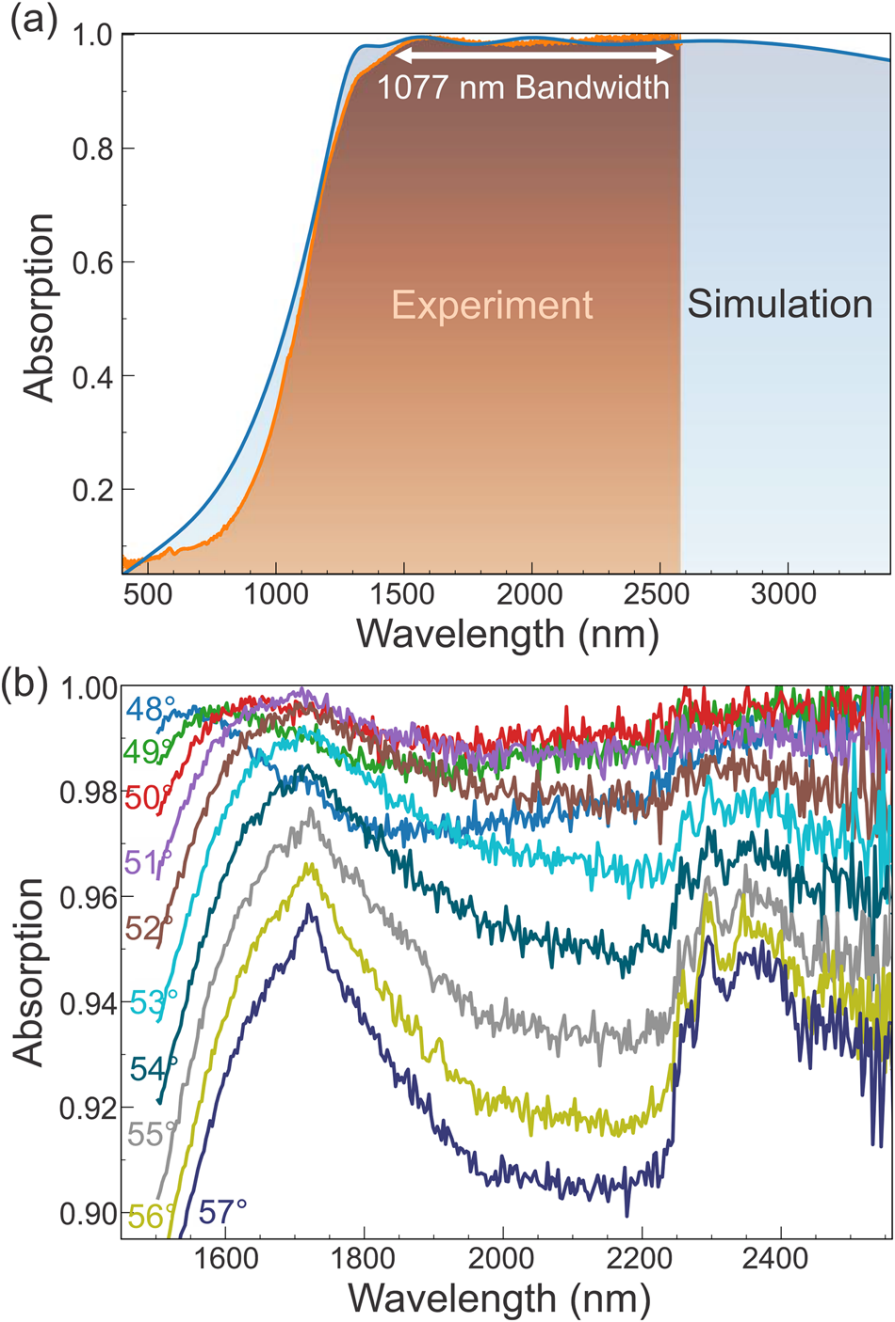
Experimental absorption measurement of the ultra‐broadband absorber. (a) The experimental measurements from the measured (orange) and simulated absorption of the neural network absorber (blue). The achieved bandwidth is 1077 nm above 98% at the design angle of 49°. (b) Zoomed‐in spectral plots of the broadband absorber at angles between 48° and 57°.

To excite the ENZ mode, we applied a phase‐matching liquid to the backside of the glass substrate and placed a BK7 prism on the backside of the substrate. Figure 3a,b shows the experimentally measured absorption, achieving a bandwidth of 1077 nm with over 98% absorption when the incident p‐polarized light inside the prism is at an angle of 49°.

The ultra‐broadband absorber exhibits such a wide absorption range that the measured absorption exceeds the detection limits of the equipment. Additionally, we also experimentally demonstrate that the ∼ 160 nm thick broadband absorber is able to achieve a bandwidth to thickness ratio of 6.86, absorbing a band approximately 7 times greater than its thickness. This result is comparable to our simulation results which shows the full bandwidth to thickness ratio to be 10.8.

To place these results in the context of absorber bandwidth limits, we compare our device With the classical Rozanov bound for multilayer absorbers [44], given by

(1) $$\frac{1}{2}\left|\ln\left(1-A\right)\left(\lambda_{\max}-\lambda_{\min}\right)\right|<2\pi^{2}\sum_{i\;=1}^{n}d_{i}$$

In Equation 1, *A* represents the threshold absorptance over the bandwidth range between λ_max_ and λ_min_, *n* is the number of layers in the stack, and d_i_ is the thickness of the individual layers of the multilayer stack. From our calculations, we find that for A=0.98 (which corresponds to 98% absorption) over a 1688 nm bandwidth, the predicted minimum thickness should be 167 nm compared to the experimental thickness of 156 nm. Therefore, when evaluated using the classical form of the Rozanov bound, our device appears to exceed the corresponding bandwidth‐thickness estimate.

The classical Rozanov limit establishes a bound on the absorption‐bandwidth product of a passive, linear, and time‐invariant slab‐based electromagnetic absorber [45], derived from the Kramers–Kronig relations. Surpassing this limit typically requires relaxing one or more of these constraints. Previous research has demonstrated such breakthroughs using active [46], time‐varying [47], and nonlinear [48, 49] systems. For instance, active absorbers incorporating non‐Foster or active impedance networks dynamically tailor their reactive and resistive components, enabling broadband absorption unattainable with passive structures. Nonlinear absorbers take advantage of intensity dependent responses to light to exceed the Rozanov limit; one example employs the use of an energy selective surface to achieve high absorption under large power incident light waves, while low absorption with low power incident in the GHz range [49]. In addition to these approaches, recent research has shown that other methods can modify this constraint, such as placing a perfectly reflecting metal backing behind the absorber or utilizing polarization [50, 51].

In our work, the deviation from the classical normal‐incidence Rozanov limit does not arise from breaking the assumptions of passivity, linearity, or time invariance. Rather, it stems from relaxing Rozanov's original assumption that absorbers must be polarization independent and at normal incidence. Under these conditions, the appropriate comparison is the relaxed bound derived by Doane et al. [52] and Firestein et al. [53], which accounts for the incidence angle for TM polarization:

(2) $$\frac{1}{2}\left|\ln\left(1-A\right)\left(\lambda_{\max}-\lambda_{\min}\right)\right|<2\pi^{2}\sum_{i\;=1}^{n}d_{i}\frac{1}{\cos\theta }$$

where θ is the angle of incidence. With this relaxed Rozanov limit, the allowed bandwidth for our absorber increases to ∼ 2400 nm at an incidence angle of 49° for our device, encompassing our simulation bandwidth of 1687 nm. Thus, the bandwidth remains within the polarization‐ and angle‐relaxed Rozanov bound. This clarifies that the observed ultra‐broadband absorption is physically allowed under the relaxed bound and is enabled by the engineered multimodal excitation of both ENZ and surface plasmon polariton (SPP) modes.

**FIGURE 4:**
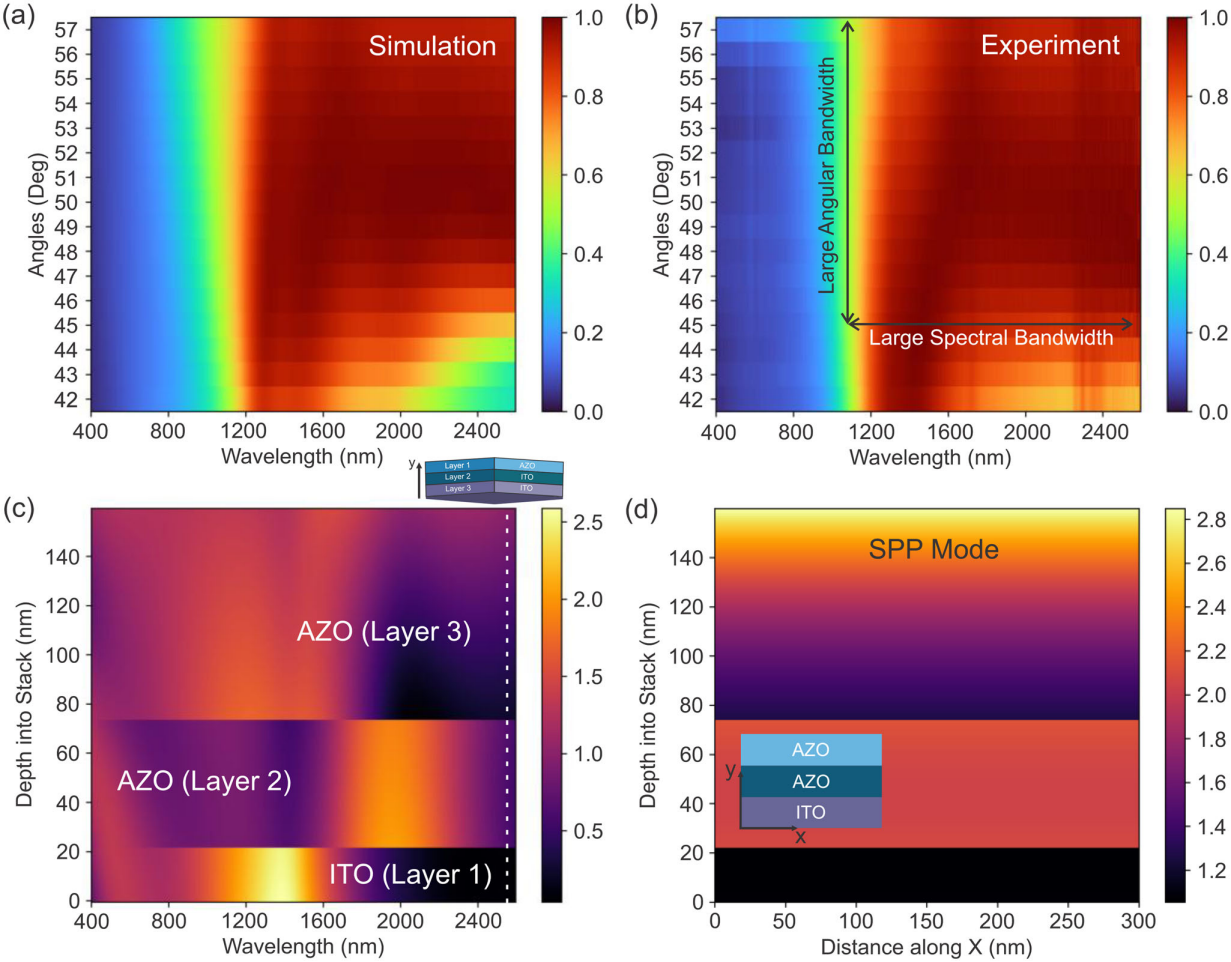
Multi‐angle measurements of the broadband ENZ absorber. (a) Simulated absorption for multiple angles from the neural network design. (b) Measured absorption of the broadband ENZ absorber. (c) Electric field magnitude of the ITO‐AZO thin film stack simulated using the experimental refractive index and thicknesses with p‐polarized light at an incident angle of 49°. White dashed line corresponds to a wavelength of 2500 nm. (d) Electric field magnitude of the same stack plotted at a wavelength of 2500 nm as a function of the horizontal position (*x*‐axis) and depth into the stack (*y*‐axis) to illustrate the SPP mode.

To validate the accuracy of our measurements and our claim of multi‐modal excitation, we compared the experimental spectra with simulations taken at multiple angles. As shown in Figure 4a,b, the two sets of results exhibit strong overall agreement. The minor discrepancies observed can be attributed to slight differences between the designed and actual ENZ wavelengths, as well as interfacial variations between the TCO layers. In addition to the strong spectral absorption, we observe a broad angular absorption bandwidth, with absorption remaining above 90% for incident angles greater than 45°. This corresponds to the critical angle of 41.8° in this wavelength range and the ENZ and SPP (surface plasmon polariton) modes that are excited above this angle. The strong agreement between the experimental and simulated results further confirms that the enhanced absorption arises from the excitation of these modes within the thin film stack.

Further investigation into mode excitation was undertaken via simulation of the electric field in the three‐layer thin film stack with rigorous coupled‐wave analysis (RCWA) modelling [54]. This is presented graphically as a function of depth within the absorber (Figure 4c). The electric field is most intense at the ENZ wavelengths of the ITO and AZO, corresponding to the ENZ mode excitation. In addition to the ENZ mode excitation, we also observe a long range (SPP) mode that propagates parallel to the film at the interface between layer 3 and air (Figure 4d). Unlike the ENZ mode, which shows a localized field enhancement in the absorbing layers at the ENZ wavelengths, the SPP appears as a localized, surface‐bound field maximum that extends laterally along the interface. This behavior appears as a bright, high‐intensity field band tightly confined along the layer‐3/air boundary, with rapid decay away from the interface and propagation parallel to the surface. The combination of both the ENZ and SPP modes effectively creates a hybrid mode that results in the ultra‐broadband absorption bandwidth of our thin film stack.

**FIGURE 5:**
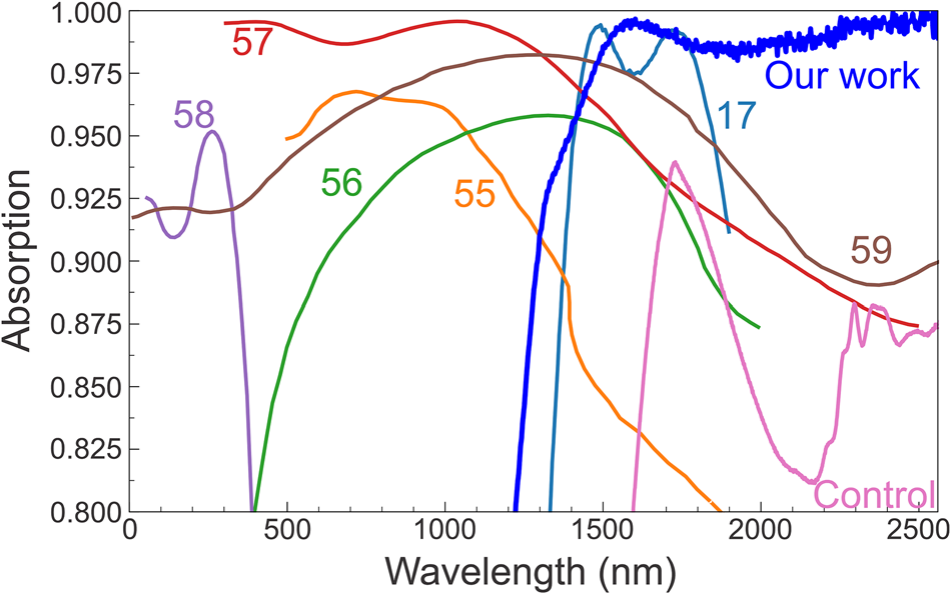
Comparison of the neural network designed broadband absorber vs. other experimental thin film and metasurface absorbers. The 159 nm‐thick neural network designed ITO‐AZO absorber is shown in dark blue. The control sample is a two‐layer film of AZO with randomly chosen ENZ wavelengths at 1450 and 2900 nm, Yoon et al. realize three layer ITO film with a total thickness of 123 nm [17], Wang et al. show a two layer film of vanadium nitride with a total thickness [55]. Seo et al. used a 400 nm metasurface, consisting of a 200 nm Cr grating covered with SiO_2_ layers [56]. Hou et al. fabricated a tapered polyimide substrate with multilayers of Ar, Cr, and SiO_2_ with a maximal thickness ∼700 nm – [57]), while Perdana et al. used a Cu nanoparticle composite layer atop a Al_2_O_3_ spacer and gold substrate, totaling a thickness of 125 nm [58]. Finally, Ren et al. validated a biologically inspired absorber which used conical‐like substrates with a height of ∼1700 nm and a ∼ 210 metal‐insulator‐metal layer on top [59].

Figure 5 and Table 1 compares our generative neural network designed absorber to other thin film absorbers and metasurfaces of similar thickness in literature. As an experimental control, we created and measured a two‐layer randomly selected AZO stack with a total thickness of 160 nm and ENZ wavelengths at 1450 and 2900 nm (80 nm thick for each layer) (see Section S4 for full deposition conditions). We emphasize that this random sample is not intended as an optimized baseline, but rather to verify if broadband absorption follows trivially from total thickness, large ENZ‐wavelength separation, or alignment of the light source to the detector. Instead, it's poor performance highlights that the absorber must be carefully optimized to achieve the desired broadband response.

**TABLE 1:** Comparison between experimentally verified absorbers and the calculated bandwidth based on the original Rozanov limit from previous works in the visible to near‐infrared regime [17, 55, 56, 57, 58, 59, 60, 61]. The numbers in paratheses represent the absorption cut‐off used to define the reported bandwidths in this table (i.e., the minimum absorption maintained over the full bandwidth, rather than wavelength‐averaged absorption). Note that due to the complex 3D geometries of the reported metasurfaces, the maximum reported structural thickness was used for the Rozanov‐limit calculations.

Type of broadband absorber	Experimental bandwidth	Rozanov maximum bandwidth	Device thickness
4 Layer ITO‐AZO Stack (Simulation) **(This work)**	1688 nm (≥ 98%)	1574 nm	156 nm
4 Layer ITO‐AZO Stack (Experiment with Detector Limitations) **(This work)**	1077 nm (≥ 98%)	1606 nm	159 nm
3 Layer ITO Stack [17]	400 nm (≥ 97%)	1384 nm	123 nm
2 Layer Vanadium Nitride/SiO_2_ [55]	1980 nm (≥ 70%)	13 116 nm	400 nm
Cr Grating [56]	1206 nm (≥ 90%)	6858 nm	400 nm
Tapered Al‐Cr‐SiO_2_‐Cr‐SiO_2_ [57]	1048 nm (≥ 98%)	6772 nm	671 nm
Copper Nanoparticles [58]	356 nm (≥ 70%)	4098 nm	125 nm
Biomimetic Gradient Resonators [59]	517 nm (≥ 98%)	19 274 nm	1910 nm
5 Layer Thin Film Stack [60]	1584 nm (≥ 90%)	7527 nm	439 nm
Nano‐cone Metamaterial Absorber [61]	2630 nm (≥ 95%)	17 988 nm	1365 nm

Focusing specifically at thin film absorbers, previous studies realized a three‐layer thin film stack of ITO with a total thickness of 123 nm that achieved a broadband absorption bandwidth of approximately 400 nm and over 97% absorption [17]. Another example is a two‐layer thin film consisting of 100 nm of SiO_2_ and 300 nm of vanadium nitride (for a total thickness of 400 nm) on top of Si substrate [55], achieving a bandwidth of 2 micron with 80% absorption threshold. Overall, these comparisons demonstrate that our generative‐design approach achieves significantly broader and higher absorption than previous thin film absorbers of comparable or even greater thickness.

## 3 Discussion

This study demonstrates the application of deep learning for metamaterial design. Using a generative neural network, we optimized a three‐layer thin film stack comprising of ITO and AZO. The optimization incorporated the Drude model and its parameters for fabricated ITO and AZO thin films in simulation. We then experimentally fabricated a broadband absorber using atomic layer deposition and RF sputtering. The fabricated absorber demonstrated an absorption bandwidth of ∼1100 nm with a subwavelength thickness of 159 nm, yielding a bandwidth‐to‐thickness ratio of approximately 7. To the best of our knowledge, this represents the broadest, experimentally demonstrated absorption bandwidth achieved by a subwavelength‐thick perfect absorber with our detector‐limited measurement range. We emphasize that simulations predict a broader absorption bandwidth, but experimental verification beyond 2600 nm is limited by the long‐wavelength cutoff of our detection system.

The ability to design ultra‐broadband, ultra‐thin perfect absorbers using deep learning unlocks new possibilities across multiple fields. In photovoltaics, these absorbers can enhance solar cell efficiency by maximizing light absorption while minimizing material use. Their broad spectral and angular absorption could also improve photodetectors, thermal imaging, radiative cooling, and thermal emission control. The high bandwidth‐to‐thickness ratio makes them ideal for compact photonic circuits and flexible optoelectronic devices, with potential applications in IR light filters, solar energy harvesting, and broadband saturable absorbers for on‐chip pulsed lasers.

Future research will explore alternative ENZ materials in the visible and near‐infrared region, such as titanium nitride and cadmium oxide, to extend absorption ranges. Further optimization of neural network architectures and hyperparameters could enhance design efficiency, while the incorporation of highly efficient grating couplers or freeform beam‐steering metasurfaces enables ENZ‐based absorbers to operate under normal‐incidence illumination. Additionally, a comprehensive benchmarking comparing various types of generative models and more recent machine learning algorithms [62, 63] represent an important direction for future work. Beyond broadband absorption, this generative approach offers a powerful tool for optimizing ENZ materials, enabling tunable ENZ devices, extreme nonlinear optics, and high‐efficiency harmonic generation for advanced laser technologies.

## 4 Materials and Methods

### 4.1 Inverse Design Framework

We selected a deep latent variable generative network, known as GLONet, since the forward method is analytic, inexpensive, and exactly differentiable with the TMM‐fast solver. This permits dataset‐free, physics‐guided training in which gradients propagate directly from the exact optical response, avoiding both the large training corpora and the surrogate‐approximation error inherent to diffusion‐based, tandem, and neural‐operator approaches, which are advantageous chiefly for computationally expensive forward solves. We emphasize that the computational efficiency demonstrated here pertains specifically to the one‐dimensional multilayer optimization considered in this work, which can be evaluated using the transfer‐matrix method. Extension of the same simulation‐in‐the‐loop framework to higher‐dimensional photonic structures requiring full‐wave electromagnetic simulations may incur substantially greater computational cost and would require additional strategies to reduce the number or expense of simulations (e.g., surrogate solvers). A quantitative comparison against gradient‐based optimization through the TMM‐Fast solver is provided in Section S2 (Figure S3).

The optimization task is framed as choosing the best carrier density (**N**), carrier mobility (**C**), effective mass (**M**), and thickness (**T**) for the layers of either aluminum doped zinc oxide [36] or indium tin oxide (ITO) to minimize the following loss function:

(3) $$\varepsilon_{i}=\;E\left[{\left(1-A\left(N,C,M,T|\lambda ,\theta \right)\right)}^{2}\right]$$

(4) $$\mathrm{Loss}\;=\frac{1}{b}\;\sum_{i=1}^{b}-exp\left(-\varepsilon_{i}\right)$$

where A(**N**, **C**, **M**, **T**|λ, θ) represents the absorption of the thin film stack and ε_*i*_ (where the index *i* denotes a specific thin film stack in the batch) represents the squared error between the thin film stack and the maximum absorption of 1, averaged over the number of wavelength points used. The loss is then computed as the negative exponential of ε_*i*_, averaged over a batch size **b**. During training, the model learns to minimize this loss function and encourages the model to generate broadband perfect absorbers.

The neural network was configured to design four‐layer thin film stacks optimized for p‐polarized light in the 400–3000 nm range (with 800 points sampled), with an incident angle of 49° inside a BK7 glass prism to excite the ENZ mode. The four‐layer design was chosen to ensure coverage of the near‐infrared region with minimal total thickness, simplifying fabrication. The Drude parameters accessible to the model were constrained by the intrinsic properties of AZO and ITO, as well as practical limits imposed by the deposition processes. ITO was deposited via radio‐frequency (RF) sputtering, while AZO was grown using atomic layer deposition (ALD). To avoid the high‐temperature annealing typically required for RF‐sputtering, the first layer was set to be ITO, while the remaining layers were restricted to AZO. Alternatively, the network can be trained to freely select either ITO or AZO in any layer, all details are provided in Sections S1 and S2.

The network was trained over 100 epochs using a batch size of 10 thin film stacks. Unlike conventional machine learning approaches that rely on pre‐generated, labeled datasets, our training process performs physics‐informed/guided training: the network generates candidate ENZ thin film stacks by sampling physical parameters, computes their absorption spectra using a physics‐based model, and backpropagates the error through the solver based on the deviation from perfect absorption.

### 4.2 Properties of Sputtered ITO and ALD Grown AZO ENZ Thin Films for Perfect Absorbers

To fabricate our generative AI‐designed broadband absorber, we employed atomic layer deposition (ALD) and radio‐frequency (RF) sputtering. ALD is a vapor‐phase technique that uses sequential, self‐limiting surface reactions to deposit atomically precise layers. The method offers nanometer‐scale control over film thickness and enables tunable doping by alternating between different chemical precursors with chamber temperature of approximately 200 °C‐250 °C. We varied two deposition parameters to change the optical properties of AZO formed in the ALD process: the ZnO‐to‐Al_2_O_3_ ratio and the chamber temperature. Decreasing the ZnO‐to‐Al_2_O_3_ ratio increases the number of Al^+3^ ions and tends to increase the carrier density. In ALD, we can control the dopant ratio by changing the number or duration of TMA or DEZ pulses. Higher temperatures typically result in a blueshift of the ENZ wavelength due to the formation of larger grain sizes. It should be noted that these relationships are nonlinear and vary significantly across different parameter ranges. Similarly, RF sputtering of indium tin oxide (ITO) allows control over Ar/O_2_ flow, RF power, and substrate temperature to tune material properties.

We fabricated a series of samples using these two deposition methods under tightly controlled conditions to match the neural network‐predicted thicknesses and optical properties. Film thicknesses and refractive indices were measured via spectroscopic ellipsometry. Figure 2c–e show ellipsometry‐derived refractive indices compared to the neural network designed targets. Using the optimized deposition parameters, we sequentially fabricated each layer of the multilayer absorber on SiO_2_‐coated glass substrates. Detailed fabrication procedures and recipes are provided in Figures S5,S6,Tables S3,S4, and S5. All layer thicknesses were within 3 nm of the designed values; measured ellipsometric angles and fitting curves are shown in Supplementary Information Figures S7a–c and S9.

## Author Contributions

DD and HWHL conceived of the project. DD and MS built the neural network architecture. DD, MS, JC, and XZ conducted numerical simulations of the structure. QD and DD performed the RF sputtering of the ITO for the sample. DD, MS, SG, JC, XZ, SL, and MA conducted the fabrication of the AZO layers with atomic layer deposition. DD, MS, JC, XZ, and MA performed ellipsometry to verify the optical properties of the AZO layers. DD performed the optical measurements with spectrophotometer. AA, WJMKK, and DD contributed to the discussion about the Rozanov bound and theory behind the absorption properties of the thin film stack. DD, WJMKK, AA, and HWHL wrote the manuscript. HWHL supervised the overall project. All authors reviewed and approved the final manuscript.

## Funding

This work was supported by the Gordon and Betty Moore Foundation (GBMF ID #13829).

## Conflicts of Interest

The authors declare no conflicts of interest.

## Supporting information




**Supporting File**: advs77966‐sup‐0001‐SuppMat.docx.

## Data Availability

The data that support the findings of this study are openly available in Figshare at https://doi.org/10.6084/m9.figshare.33970843.
