# Supplementary material for: Generative AI‐Enabled Discovery and Experimental Demonstration of Ultra‐Broadband Epsilon‐Near‐Zero Perfect Absorbers
Source: Adv Sci (Weinh). 2026 Sep 27:e77966. Online ahead of print. doi: 10.1002/advs.77966 (PMC13616347; doi:10.1002/advs.77966)
Supplement: Supplementary file 1 [file ADVS-9999-e77966-s001.docx]

**Supporting Information**

**Generative AI-Enabled Discovery and Experimental Demonstration of Ultra-Broadband Epsilon Near Zero Perfect Absorbers**

David Dang^1,2,3,4,5^, Meena Salib^1^, Quynh Dang^1,3,4^, Sudip Gurung^1^, Juan Calixto^1^, Xuguo Zhou^1^, Stuart Love^1,3,4^, Massee Akbar^1,3,4^, Aleksei Anopchenko^1,3,4^, Wilton J.M. Kort-Kamp^5^, and Ho Wai Howard Lee^1,3,4*^

^1^Department of Physics and Astronomy, University of California Irvine, CA 92697, USA.

^2^Center for Integrated Nanotechnologies, Los Alamos National Laboratory, NM 87545, USA.

*^3^Beckman Laser Institute and Medical Clinic, University of California, Irvine, CA 92697, USA*

*^4^Eddleman Quantum Institute, University of California, Irvine, CA 92697, USA*

^5^Theoretical Division, Los Alamos National Laboratory, NM 87545, USA.

*Corresponding author: [howardhw.lee@uci.edu](mailto:howardhw.lee@uci.edu)

**Supplementary Section 1: Neural Network Training**

**Figure S1: Generative Neural Network Training and Architecture: (a)** Training performance of the neural network via observation of the loss function versus number of batch (or iterations). Lower values indicate better performance. **(b)** Loss values compared with different number of network blocks (i.e. increasing the size of the model). **(c)** Neural network architecture of the residual neural network blocks.

To enable the inverse design of multilayer optical structures, we implement a generative neural network conditioned on physically meaningful parameters: **carrier concentration**, **mobility**, **thickness**, and **material type**- Aluminum Doped Zinc Oxide (*1*) vs Indium Tin Oxide ITO (ITO). These parameters are discretized over experimentally relevant ranges and sampled probabilistically using a softmax-based selection process.

***Parameter Encoding and Input Representation***

Prior to training, the design space is discretized across a grid of physically relevant values for both AZO and ITO layers:

- **Carrier concentration**: defined over a range [𝑛ₘᵢₙ, 𝑛ₘₐₓ] with step Δ𝑛
- **Mobility**: defined over a range [μₘᵢₙ, μₘₐₓ] with step Δμ
- **Layer thickness**: defined over [𝑡ₘᵢₙ, 𝑡ₘₐₓ] with step Δ

Each layer in the stack is modeled as a categorical selection over these predefined values. During training, a random latent code **𝑧** is sampled from a normal distribution and passed through the generator network.

***Softmax Selection with Temperature Scaling***

The generator outputs a tensor containing **unnormalized logits** for each parameter dimension (e.g., 𝜔ₚ, γ, thickness), which are then transformed into **probability distributions** using the softmax function:

$$(1.1 ) P\left( x_{i} \right)=\frac{\exp\left( \alpha\cdot x_{i} \right)}{\sum_{j} \exp\left( \alpha\cdot x_{j} \right)}$$

Here, **α** is a temperature-like scaling factor that is **annealed over training iterations**. Initially, low α results in nearly uniform distributions (exploration), while higher α encourages peaky distributions (exploitation), forcing the model to converge toward deterministic parameter selections.

The final parameter values are computed as **expected values** over the discrete grids:

$$(1.2) \text{value}_{\text{eff}}=\sum_{i} P\left( x_{i} \right)x_{i}$$

This process is repeated for each layer and each parameter type (e.g., thickness, ωₚ, γ). Additionally, a two-way softmax is applied to determine whether each layer should be AZO or ITO. For the main manuscript, the probability values of whether each layer should be AZO or ITO, is manually set such that the first layer will always be ITO and the subsequent layers will be AZO.

In the main text, we implemented a residual generative neural network comprising residual neural network blocks. As illustrated in Figure S1a, the loss function stabilizes at a horizontal plateau after approximately 40 iterations. Figure S1b demonstrates that deeper network architecture show little to no change as we increase the size from 3 to 16 residual neural network blocks and indicates that the model is a sufficiently large size to avoid underfitting the design space.

Each block includes the following layers: an input layer with 512 neurons, a fully connected layer with 1024 neurons, batch normalization, a leaky ReLU activation function (slope parameter 0.2), a subsequent fully connected layer with 512 neurons, and a final layer with of batch normalization (Fig. 1c). The total number of trainable parameters for the neural network is **13,477,528**. Despite its size, the network converges efficiently within a reasonable number of iterations. We used an Intel 12^th^ Gen Core i5-12600K CPU and an Nvidia RTX 3090 GPU for running our neural network model. Each iteration processes a batch of 10 thin film stacks, with their losses and gradients averaged for backpropagation during mini-batch gradient descent.

**Table S1. Parameters that the neural network can choose for the ITO and AZO layers.** These parameters are consistent with previously reported values of fabricated ITO and AZO*(2-9)*. A lower bound of **10 nm** was selected for the film thickness, as this was the minimum value at which the optical properties of the fabricated thin films could be reliably determined. The parameter space was sampled with a **1 nm step size** for thickness, a **0.2 × 10²⁰ cm⁻³ step size** for carrier density, and a **1 cm² V⁻¹ s⁻¹ step size** for carrier mobility.

The optical properties of ITO/AZO is described by free-electron Drude model^(^*^10^*^)^:

(1.3)	$\varepsilon\left( \text{ω} \right)=\varepsilon_{\infty}-\frac{\omega_{p}^{2}}{\text{ω}^{\text{2}}+\text{iωΓ}}$	where	$\text{ω}_{\text{p}}=\sqrt{\frac{\text{N}e^{2}}{\varepsilon_{0}m^{*}}}$

*ε_∞_*, *ω_p,_* and Г represent the permittivity at high frequency limit, plasma frequency, and electron collision rate, respectively. The plasma frequency, *ω_p_*, is a function of the electron density N, electron charge *e*, permittivity of free space *ε_0_*, and effective electron mass *m^*^*. The value of *є_∞_* is set to 3.9 for both ITO and AZO.

**Table S2:** Comparison of past(*11*) and present approaches for ENZ multilayer absorbers.

**Supplementary Section 2: Additional Simulations**

As mentioned in the main text, the model can assign AZO or ITO for each layer in the multilayer thin film stack, but we restricted the first layer to be ITO and subsequent layers as AZO to prevent annealing of the AZO layers. To evaluate the performance of the model with a greater degree of freedom, we retrained our model and generated another stack of 10,000 thin films with unrestrained layer assignments.

The results of this new optimization are shown in Figure S2, which shows a broadband absorber with a large broadband absorption bandwidth also greater than 1600 nm. These results confirm that the proposed framework is capable of jointly optimizing both material assignment and layer thickness when fabrication constraints are removed, and that the fixed material sequence adopted in the main text was solely a fabrication-driven design choice.

**Figure S2: Unrestricted Model Generation** (a) Bandwidth histogram of generated devices (mean: 383 nm, Std: 265 nm). (b) Absorption plot of best performing absorber with a bandwidth of 1647 nm, similar in bandwidth to our fabricated absorber. (c) Refractive index, thickness values, and material assignments of the unrestricted broadband absorber. Here, the third layer is chosen as ITO.

To benchmark the neural-network-generated designs against a non-deep-learning optimization baseline, we performed direct gradient-based optimization using the differentiable TMM solver. Specifically, 10,000 randomly initialized devices were optimized for 40 gradient descent iterations, corresponding to 400,000 total TMM simulations (Fig. S3). Despite this substantially larger simulation budget, the gradient-based optimization baseline significantly underperformed relative to the neural-network-based design approach, which used 20,000 total simulations for training and evaluation.

The maximum absorption bandwidth obtained by gradient descent was approximately 608 nm above the 98% absorption threshold, nearly three times smaller than the best neural-network-generated design. In addition, for devices that reached the 98% absorption threshold, the average bandwidth was approximately 94 nm for gradient descent, compared with approximately 341 nm for the neural-network-generated designs. These results indicate that, even within this relatively low-dimensional multilayer design space, direct gradient-based optimization was less effective at identifying ultra-broadband absorber structures under the same material and structural constraints.

**Figure S3: Gradient-Based Optimization: (a)** Bandwidth histogram of devices generated by gradient descent . **(b)** Absorption plot of the best performance thin film stack generated by gradient descent.

To assess the effect of merging the third and fourth layers, we performed additional simulations comparing the original four-layer design with a modified three-layer stack (Fig. S4). In the three-layer structure, the final layer retained the refractive index of the original third layer, while its thickness was increased to 88 nm by combining the original third- and fourth-layer thicknesses. The resulting absorption spectrum showed only minor changes relative to the original design, with a 98% absorption bandwidth of 1634 nm, corresponding to an approximately 3.2% difference. The peak absorption also increased slightly from 99.60% to 99.74%, indicating that the simplified three-layer structure preserves the broadband absorption behavior of the neural-network-generated design.

**Figure S4: Original Network Absorber (4 layer) vs 3 Layer Merged Absorber:** Absorption spectrum of the Absorption spectrum of the four-layer (blue line) and three-layer absorber (black dashed line).

**Supplementary Section 3: Fabrication of Neural Network Optimized Broadband Absorber**

For the deposition of AZO, three precursors are required: trimethylaluminum (TMA, Al(CH_3_)_3_), diethylzinc (DEZ, Zn(C_2_H_5_)_2_), and water. The deposition cycle is explained as follows(*12-16*):

2.1 a: ||- OH + Al(CH_3_)_3_ 🡪 ||-O-Al (CH_3_)_2_ + CH_4_

2.1 b: ||-O-Al (CH_3_)_2_ + OH 🡪 ||-O_2_-Al(CH_3_) + CH_4_

2.2: ||-O_2_-Al(CH_3_) + H_2_O 🡪 ||-O_2_-Al-OH + CH_4_

2.3: ||- OH + Zn(C_2_H_5_)_2_ 🡪 ||-O-Zn(C_2_H_5_) + C_2_H_6_

2.4: ||-O-Zn(C_2_H_5_) + H_2_O 🡪 ||-OZn-(OH) + C_2_H_6_

We use a benchtop ALD deposition system by GEMSTAR with 98% purity TMA and 95% purity DEZ from Strem Chemicals.

For deposition of ITO, we use RF sputtering; a process whereby radio-frequency waves are used to create a plasma of argon inert gas, causing the positive argon ions to bombard a negatively charged ITO target (Kurt J. Lesker In2O3/SnO3 90/10 wt%, 99.99% pure, indium bonded to copper back plate), ejecting ITO atoms onto a heated substrate. The high temperatures (500-900 C) involved in RF-sputtering limit the ITO layer to only the first layer of our absorber to avoid annealing of the AZO layers. Single side polished Si substrates with ~ 2 nm of SiO_2_ on top were used. Before each deposition, we cleaned the substrates by submerging them in an ultrasonic acetone bath for approximately 1 minute and then an ultrasonic isopropyl alcohol bath for another minute then rinsed with deionized water and dried with compressed air (Fig. S5).

**Figure S5: Process for the fabrication of the multi-thin film stack absorber:** After ITO sputtering and AZO ALD, the backside of the SiO_2_ glass substrate will have a buildup of material due to the gaseous nature of the ALD precursors. We tape the front side with Teflon tape and use a pipette to place a few drops of sulfuric acid (98%) to clean the backside. After washing off excess acid with water, we measure the resistance on both sides to verify that our cleaning procedure worked. Due to the conductivity of AZO, we expect to see much less resistance on the frontside than the backside of the glass.

Listed below are the fabrication recipes for the broadband absorber using RF sputtering (Table S3) and ALD (Table S4, Table S5).

**Table S3: RF Sputtering Recipe for ITO.** The table summarizes the key process parameters used for radio-frequency ITO deposition on the Orion-8 UHV Sputtering System.

**Table S4: Atomic Layer Deposition Recipe for the 1^st^ AZO Layer**. Note that after each pulse duration a 40 millisecond (mSec) delay was included.

**Table S5: Atomic Layer Deposition Recipe for the 2^nd^ AZO Layer.** Note that after each pulse duration a 40 millisecond (mSec) delay was included similar to the previous recipe.

The ALD fabricated samples on Si of the neural network designed broadband absorber are shown in Figure S6. To characterize the optical properties of our samples, we performed spectroscopic ellipsometry and fitted the measured psi and delta using the Drude model. Figure S7 shows close agreement between the fitting values and the measured psi and delta. This indicates that the fabrication process successfully produced the intended optical properties as well as validating the extracted refractive index of our fabricated layers.

**Figure S6: Individually Fabricated Layers of AZO on Silicon.** The deposition of the materials on Si allows it to be visible. The brown sample (left side) is the 1^st^ AZO layer, while the blue sample (right side) is the 2^nd^ AZO layer.

**Figure S7: Psi and Delta fitting from ellipsometry for each layer of the neural network designed broadband absorber.** The maximum wavelength range of the ellipsometer is limited to 1750 nm but was extrapolated to 3000 nm for the refractive index using the Drude model parameters obtained from ellipsometry.

**Supplementary Section 4: Absorption Measurement of Broadband Absorber**

For measuring the absorption of the broadband absorber, we used the Cary-7000 Universal Spectrophotometer (Fig. S8). This grating-based spectrophotometer allows precise selection and measurement of individual wavelengths across a broad spectral range, with the additional capability of controlling the polarization state of the incident light. By measuring the transmitted and reflected light, we were able to calculate the absorption of the samples across the relevant wavelength range, providing a comparison with simulation results, enabling validation of both the design and fabrication processes.

**Figure S8: Diagram showcasing the measurement of the broadband absorber with the Cary Universal Spectrophotometer.** The detector and stage are both able to rotate, allowing the spectrophotometer to collect transmission and reflection for the absorption calculations.

**Supplementary Section 5: Control Multilayer Thinfilm Stack**

**Figure S9: Refractive Index of the Randomized Broadband/Control Sample:** The real part of the refractive is given in the solid line, while the imaginary part is the dashed line. Both layers were measured to be approximately 80 nm via spectroscopic ellipsometry.

As an experimental control, we fabricated a two-layer absorber, with randomly selected ENZ resonances, but same total thickness (80 nm for each layer for a total thickness of 160 nm) (Fig. S9). With our control sample, we wanted to demonstrate that the high absorption threshold and bandwidth was not simply due to the total thickness of the sample or issues with our optical setup, but rather careful engineering of the geometry and optical properties of the layers. See Table S6 and S7 for the ALD recipes of the control samples’ layers.

**Table S6: Atomic Layer Deposition Recipe for the 1^st^ AZO Layer of the Control Sample.** Note that after each pulse duration a 40 millisecond (mSec) delay was included.

**Table S7: Atomic Layer Deposition Recipe for the 2^nd^ AZO Layer of the Control Sample.** Note that after each pulse duration a 40 millisecond (mSec) delay was included.

**References**

1.	Z. Omair *et al.*, Ultraefficient thermophotovoltaic power conversion by band-edge spectral filtering. *Proc Natl Acad Sci U S A* **116**, 15356-15361 (2019).

2.	R. Bi, C. Zheng, W. W. Yu, W. Zheng, D. Wang, Breaking through the plasma wavelength barrier to extend the transparency range of ultrathin indium tin oxide films into the far infrared. *Journal of Applied Physics* **134**, (2023).

3.	S. F. Blair, J. S. Male, S. A. Cavill, C. P. Reardon, T. F. Krauss, Photonic characterisation of indium tin oxide as a function of deposition conditions. *Nanomaterials* **13**, 1990 (2023).

4.	J. W. Cleary, E. M. Smith, K. D. Leedy, G. Grzybowski, J. Guo, Optical and electrical properties of ultra-thin indium tin oxide nanofilms on silicon for infrared photonics. *Optical Materials Express* **8**, 1231-1245 (2018).

5.	X. Liu *et al.*, Quantification and impact of nonparabolicity of the conduction band of indium tin oxide on its plasmonic properties. *Applied Physics Letters* **105**, (2014).

6.	S. Gurung *et al.*, Atomic Layer Engineering of Epsilon‐Near‐Zero Ultrathin Films with Controllable Field Enhancement. *Advanced Materials Interfaces* **7**, 2000844 (2020).

7.	A. Anopchenko, L. Tao, C. Arndt, H. W. H. Lee, Field-effect tunable and broadband epsilon-near-zero perfect absorbers with deep subwavelength thickness. *ACS Photonics* **5**, 2631-2637 (2018).

8.	H. Fujiwara, M. Kondo, Effects of carrier concentration on the dielectric function of ZnO: Ga and In 2 O 3: Sn studied by spectroscopic ellipsometry: Analysis of free-carrier and band-edge absorption. *Physical Review B* **71**, 075109 (2005).

9.	J. Zhang *et al.*, Gate-tunable optical filter based on conducting oxide metasurface heterostructure. *Optics Letters* **44**, 3653-3656 (2019).

10.	P. Drude, Zur elektronentheorie der metalle. *Annalen der physik* **306**, 566-613 (1900).

11.	D. Dang *et al.*, Neural network design of broadband epsilon near zero perfect optical absorbers. *Journal of Materials Chemistry C*, (2023).

12.	G. P. Gakis *et al.*, Investigation of the initial deposition steps and the interfacial layer of Atomic Layer Deposited (ALD) Al2O3 on Si. *Applied Surface Science* **492**, 245-254 (2019).

13.	J. Cai *et al.*, A revisit to atomic layer deposition of zinc oxide using diethylzinc and water as precursors. *Journal of Materials Science* **54**, 5236-5248 (2019).

14.	E. Riyanto *et al.*, A review of atomic layer deposition for high lithium-ion battery performance. *Journal of Materials Research and Technology* **15**, 5466-5481 (2021).

15.	E. Guziewicz *et al.*, Zinc Oxide Grown by Atomic Layer Deposition: From Heavily n‐Type to p‐Type Material. *physica status solidi (b)* **257**, 1900472 (2020).

16.	G. P. Gakis *et al.*, Detailed investigation of the surface mechanisms and their interplay with transport phenomena in alumina atomic layer deposition from TMA and water. *Chemical Engineering Science* **195**, 399-412 (2019).
